# Special Issue: “Molecular Imaging in Oncology: Radiopharmaceuticals for PET and SPECT 2022”

**DOI:** 10.3390/ph17010049

**Published:** 2023-12-28

**Authors:** Junbo Zhang

**Affiliations:** Key Laboratory of Radiopharmaceuticals of Ministry of Education, NMPA Key Laboratory for Research and Evaluation of Radiopharmaceuticals (National Medical Products Administration), College of Chemistry, Beijing Normal University, Beijing 100875, China; zhjunbo@bnu.edu.cn; Tel.: +86-10-6220-8126

Molecular imaging is partly defined as in vivo imaging of biological or biochemical processes using various markers. It can realize early diagnosis of disease and evaluation of therapeutic response [1]. The widespread utilization of molecular biology techniques, coupled with the rapid advancement of radionuclide tracing technology such as positron emission tomography (PET) and single photon emission computed tomography (SPECT), has ushered in a new era in the field of molecular nuclear medicine. Radiopharmaceuticals that are administered to the patient can significantly localize to the target tissue. Sensible utilization of tailored radiopharmaceuticals offers a valuable means to investigate the molecular and cellular mechanisms that underlie the progression of diseases in a safe and non-invasive manner.

Cancer has been one of the major causes of death worldwide. Numerous researchers in the fields of radiochemistry, nuclear medicine professionals, and nuclear physicists have dedicated significant efforts to developing more effective diagnostic and therapeutic tumor radiopharmaceuticals while also making strides in the advancement of synthesis modules to facilitate practical applications. The topics chosen for the Special Issue mainly focus on the development of novel radiopharmaceuticals, especially those dedicated to tumor imaging and therapy.

Prostate-specific membrane antigen (PSMA) is an ideal target for both diagnosing and treating prostate cancer [2]. In this Special Issue, Ren et al. introduced a novel PSMA tracer called [^64^Cu]Cu-PSMA-CM. This tracer was modified with maleimidopropionic acid (MPA) to enhance its uptake in PSMA-positive tumors. The tracer exhibited favorable physicochemical and biological properties, including a prolonged half-life in the bloodstream and a strong affinity to PSMA. These characteristics facilitated the monitoring of radioactivity accumulation in PSMA-targeted tracers within tumors.

Wu et al. modified the structure of PSMA-617 by replacing the naphthyl group with three different groups: 4-pyridyl (4PY), 3-quinoline (Q), and pyrene (P). Among these compounds, [^68^Ga]Ga-PSMA-Q showed a higher tumor-to-muscle ratio (59.33 ± 5.72 at 60 min p.i.) compared to [^68^Ga]Ga-PSMA-617. Further modifications were made to the linker group of the selected compound (PSMA-Q) with propyl (PSMA-Q-1), butyl (PSMA-Q-2), and phenyl (PSMA-Q-3) to decrease hydrophilicity and enhance tracer accumulation. However, the results consistently indicated that PSMA-Q remained the most suitable option. Due to its favorable tumor-to-background ratio, PSMA-Q holds great potential for future treatment when labeled with other therapeutic nuclides.

Programmed cell death protein 1 (PD-1) and its ligand (PD-L1) immune checkpoint inhibitors are the current research hotspots of tumor immunotherapy [3]. These inhibitors have shown remarkable therapeutic effects on various types of tumors. However, the conventional method of detecting PD-L1 expression through immunohistochemistry (IHC) is invasive and lacks real-time monitoring capabilities. Xu et al. devised a novel imaging agent called [^18^F]LP-F, which contains a polyethylene glycol (PEG) moiety and is produced through a simple one-step ^18^F-fluorination process. This agent specifically accumulates in cells and tumors with high PD-L1 expression within just 30 min. Moreover, [^18^F]LP-F exhibits a sustained and relatively higher target-to-muscle (T/M) ratio in PD-L1 (+) tumors compared to PD-L1 (−) tumors, indicating its potential to differentiate between tumors with varying levels of PD-L1 expression.

Another report from Xiang et al. focused on peptide-based pharmaceuticals. They modified a peptide called WL12, which targets PD-L1, by incorporating HBED-CC as the coupling group and labeling it with ^68^Ga. [^68^Ga]Ga-WL12 exhibited excellent quality within the initial 20-min administration and continued to accumulate in the tumors for up to 180 min. The highest tumor-to-liver (T/L) ratio of 0.45 ± 0.03 was achieved at 90 min post-injection, while the tumor-to-muscle (T/M) ratio exceeded 2 after 90 min. These findings suggest that [^68^Ga]Ga-WL12 can serve as a valuable tool for guiding clinical treatments.

Compared to expensive and limited PET imaging, SPECT imaging is a more cost-effective and widely utilized imaging technique. ^99m^Tc has excellent radionuclide properties, including a proper half-life, gamma-ray energy of 140 keV, and the awesome capability of chelating, making it the most commonly used radionuclide in SPECT for clinical applications. Fibroblast activator protein (FAP) is selectively expressed in cancer-associated fibroblasts (CAFs) in the stroma of most solid tumors, making it a potential target for tumor diagnosis and treatment [4]. In this Special Issue, Luo et al. designed a compound called [^99m^Tc]Tc-HYNIC-FAPI-04, which utilizes 6-hydrazinonicotinate-aminocaproic (HYNIC) as a bifunctional chelating ligand. SPECT/CT imaging showed a high uptake of [^99m^Tc]Tc-HYNIC-FAPI-04 in the U87MG tumor (2.67 ± 0.35 %ID/mL at 1.5 h p.i.), while the FAP-negative HUH-7 tumor had a low uptake (0.34 ± 0.06 %ID/mL at 1.5 h p.i.). At 5 h post-injection, the U87MG tumor showed higher tumor-to-liver ratios, allowing for longer observation of tumor status.

Excessive proliferation leads to a greater demand for glucose in cancer cells compared to normal cells [5]. Glucose is utilized as the core structure of many imaging drugs, including the highly regarded 2-deoxy-2-[^18^F]fluoro-D-glucose ([^18^F]FDG), which is widely employed in clinical settings. Feng et al. synthesized a glucose derivative containing cyclohexane and isonitrile (CNMCHDG) and labeled it with ^99m^Tc using a simple kit formulation. Biodistribution and SPECT imaging studies showed significant accumulation of [^99m^Tc]Tc-CNMCHDG in A549 tumor-bearing mice (4.42 ± 0.36 %ID/g at 120 min p.i.) with good retention. Moreover, [^99m^Tc]Tc-CNMCHDG exhibited excellent tumor-to-non-target ratios, a clear imaging background, and holds promise as a potential candidate for clinical translation.

Therapeutic radiopharmaceuticals undergo selective and targeted treatment of diseased tissue by radionuclides. A comprehensive review by Liu et al. studies three therapeutic methods. Transarterial radioembolization (TARE) can extend disease-free survival through a hepatic artery radiopharmaceutical injection. Although ^90^Y-microsphere TARE shows great promise as a routine treatment practice, there are still uncertainties regarding its prognostic effect in different patients. On the other hand, ^125^I seed implantation and ^131^I-metuximab radioimmunotherapy for HCC have gained increasing attention, but their efficacy needs to be validated through further randomized controlled trials.

Gold nanoparticles (AuNPs) possess the ability to be easily synthesized and modified with various biomolecules and anticancer drugs. In this Special Issue, Mhanna et al. have successfully produced two types of dual-ligand AuNPs, namely PEG-MTX-AuNP and MTX-RGD-AuNP. Mhanna et al. employed a chelator-free ^64^Cu labeling method, creating ^64^Cu-labeled AuNP conjugates that exhibit significantly improved in vivo stability. Both in vitro and in vivo studies have demonstrated that the uptake of dual-ligand AuNPs by tumors surpasses that of single-ligand AuNPs.

Radiosynthesis modules are automated pieces of equipment utilized in molecular labeling. They play a crucial role in the development and manufacturing of PET radiotracers. In this Special Issue, Waśniowski et al. conducted the automated synthesis of [^18^F]F-DOPA and its subsequent modifications using the ABX 1336 precursor in conjunction with the Raytest SynChrom R&D module. To prevent the accumulation of impurities after synthesis, a module equipped with disposable radiosynthesis cassettes was employed immediately after the synthesis process. The synthesis procedure lasted for 120 min, resulting in a radiochemical yield of 15% and a radiochemical purity of at least 97%. These outcomes demonstrate a highly efficient and pure advancement in radiosynthesis.

Overall, many achievements on novel PET and SPECT radiopharmaceuticals for molecular imaging have been made. However, great challenges still lie ahead before more effective tumor radiopharmaceuticals for molecular imaging go into clinical trials. We extend our sincere gratitude to all the authors, reviewers, and editors for their invaluable contributions, and I hope readers will enjoy this Special Issue.
